# Label-free fluorescent aptasensor for chloramphenicol based on hybridization chain reaction amplification and G-quadruplex/*N*-methyl mesoporphyrin IX complexation†

**DOI:** 10.1039/d2ra00572g

**Published:** 2022-06-22

**Authors:** Wentao Zheng, Yubin Li, Liting Zhao, Ciling Li, Lei Wang

**Affiliations:** Zhanjiang Central Hospital, Guangdong Medical University Zhanjiang 524045 China wangleigdmu@163.com; Faculty of Chemistry & Environmental Science, Guangdong Ocean University Zhanjiang 524088 China

## Abstract

The use of the broad-spectrum antibiotic chloramphenicol (CAP) in food is strictly regulated or banned in many countries. Herein, for the sensitive, rapid, and specific detection of CAP in milk, a label-free fluorescence strategy was established based on guanine (G)-quadruplex/*N*-methyl mesoporphyrin IX (NMM) complex formation and hybridization chain reaction (HCR) amplification. In this system, CAP can specifically bind to an aptamer (Apt) to release an Apt-C sequence from double-stranded DNA (Apt·Apt-C). Apt-C, can further hybridize with a functional hairpin DNA probe to release a primer sequence. The released primer sequence causes HCR and the formation of a nicked double-helix polymer, which contains G-quadruplex DNA. The recognition of G-quadruplex DNA by the NMM fluorochrome results in fluorescence enhancement. Consequently, CAP can be quantitatively detected by measuring the fluorescence intensity at 612 nm. The reliability of the aptasensor method was confirmed by comparison with an enzyme-linked immunosorbent assay. The proposed aptasensor was found to have a limit of detection of 0.8 pg mL^−1^ for CAP. Moreover, when the aptasensor was applied to the detection of CAP in milk samples, the average recoveries were 99.8–108.3% with relative standard deviations of 4.5–5.2%. Thus, this CAP detection method, which is rapid with high sensitivity and selectivity, has considerable potential for a wide range of food analysis applications.

## Abstract 2

For the sensitive and specific detection of CAP in milk, a label-free fluorescence strategy was established based on guanine (G)-quadruplex/*N*-methyl mesoporphyrin IX (NMM) complex formation and hybridization chain reaction (HCR) amplification.

## Graphical abstract

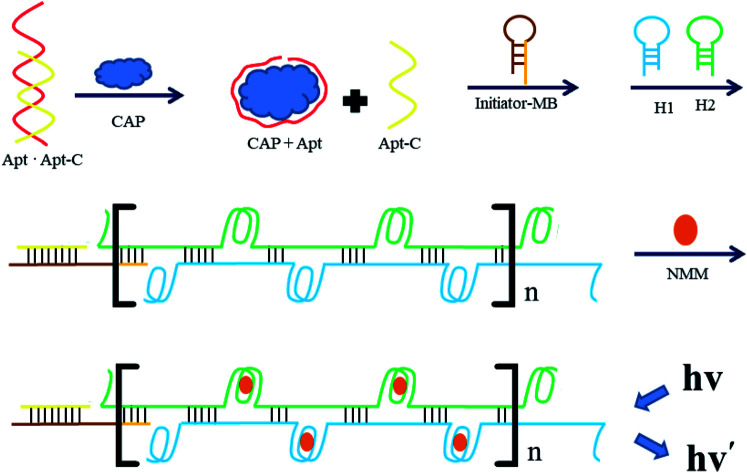

## 1. Introduction

Chloramphenicol (CAP) is a bacteriostatic broad-spectrum antibiotic that is effective against Gram-negative and Gram-positive bacteria.^1,2^ CAP is broadly used in humans, poultry, and aquatic species to control and treat infectious diseases owing to its remarkable antibacterial effects and low cost.^3,4^ Nevertheless, the enrichment of CAP residues in the human body through food-borne bioaccumulation can have significant health effects, including leukemia,^5^ gray baby syndrome,^6,7^ and aplastic anemia.^8,9^ Hence, the use of CAP in food-producing animals has been strictly regulated or banned in many countries. The European Union has set the maximum allowed performance limit of CAP at 0.3 μg kg^−1^ in various foods.^10–12^

Various analytical techniques have been employed for CAP determination, such as surface-enhanced Raman scattering,^13,14^ liquid chromatography–mass spectrometry^15,16^ and high-performance liquid chromatography–mass spectrometry.^17,18^ Although these traditional analytical methods are sensitive and accurate, they have certain limitations, as they require costly instruments, tedious operational procedures, time-consuming sample pretreatment, and well-trained technicians. Consequently, the development of rapid, accurate, and sensitive methods for the detection of CAP *in vitro* and in real food samples remains of practical significance to protect food safety and public health.

An aptamer is a DNA or RNA oligonucleotide (primarily single-stranded) that can specifically bind a target molecule with excellent affinity. Aptamers are typically screened using the systematic evolution of ligands by exponential enrichment (SELEX) technique.^19–21^ As biorecognition elements, aptamers are low cost, easy to synthesize and modify, and have better targeting properties than antibodies.^22,23^ Recently, numerous aptasensors for CAP monitoring have been reported based on fluorescent biosensors,^24,25^ colorimetry,^26,27^ and electrochemical methods.^28,29^ Among these proposed methods, fluorescent biosensors are considered promising owing to their simplicity, sensitivity, and specificity. However, most fluorescent aptasensors were formed of labeled arrays. The binding affinity between the aptamer and the target will decrease with the attachment of the label, thereby affecting the sensitivity of the aptasensor. Compared with the labeled approaches, the label-free fluorescent aptasensor has low cost, simple operation, and better reproducibility.

To improve the sensitivity of biosensors, various methods based on signal amplification strategies have been rapidly developed, including hybridization chain reaction (HCR) amplification,^30,31^ polymerase chain reaction amplification,^32,33^ rolling circle amplification,^34,35^ and strand displacement amplification.^36,37^ Among these strategies, HCR is considered to have great potential for signal amplification owing to its enzyme-free nature, simplicity, economy, isothermality, and excellent sensitivity. The HCR method has been extensively used in the determination of oligonucleotides,^38^ proteins,^39^ and tumor cells.^40^

Guanine (G)-quadruplexes are high-order structures consisting of guanine-rich DNA or RNA sequences.^41,42^*N*-Methyl mesoporphyrin IX (NMM) is an unsymmetrical anionic porphyrin with remarkable structural selectivity for G-quadruplexes over triplexes, double-stranded oligonucleotides, or single-stranded oligonucleotides.^43,44^ As complexation results in a significant fluorescence enhancement, G-quadruplex/NMM complexes have found wide application in the fabrication of diverse biosensors with high affinities for the determination of DNA, small molecules, and metal ions.^45–49^ The fluorescence enhancement caused by the complexation reaction of NMM with G-quadruplexes was used for target substance detection. Not necessary to add enzymes to the catalytic activity through enzyme cascade reaction, but also without the DNA immobilized on the electrode, which avoids the complicated process such as labeling and the influence of additional media. Consequently, it has the advantages of simplicity, low cost, and rapidity.

Herein, a label-free fluorescence strategy for the sensitive detection of CAP was established based on HCR amplification and G-quadruplex/NMM complexation. In the absence of CAP, the HCR couldn't be triggered, and there were hardly any signals that could be found. In contrast, upon the addition of CAP, the complementary strand was released from dsDNA, which subsequently hybridized with the initiator to release the primer sequence. The primer sequence triggered the HCR to initiate a hybridization cascade that forms a nicked double-helix polymer. The formation of the HCR product with stable G-quadruplex DNA structures can be monitored using the fluorescence of the G-quadruplex-specific fluorochrome NMM.^50^ Accordingly, the quantitative analysis of CAP was achieved based on the increase in the fluorescence intensity at 612 nm.

## 2. Materials and methods

### 2.1. Materials and reagents

Oligonucleotides were purchased from Sangon Biotech Co., Ltd (Shanghai, China). All the sequences (Table S1†) were dissolved in Tris-HAc buffer (50.0 mmol L^−1^, pH 7.9, 50.0 mmol L^−1^ KAc). CAP, cefixime, ciprofloxacin, tetracycline, amoxicillin, and spermine were purchased from Sinopharm Chemical Reagent Co., Ltd (Shanghai, China). NMM was purchased from J&K Scientific Ltd (Beijing, China). A CAP enzyme-linked immunosorbent assay (ELISA) kit was obtained from Beacon Analytical Systems, Inc. (Saco, MA, USA). Milk samples were obtained from a supermarket in Zhanjiang city (Yili Corporation, Inner Mongolia, China). Ultrapure water (18.1 MΩ cm, 25 °C) was obtained using a 350 Nanopure water system (Guangzhou, China) and was utilized in all experiments. All reagents were of analytical grade.

The two hairpin DNA probes (H1 and H2) and the initiator probes were heated at 90 °C for 10 min and then slowly cooled to room temperature to form satisfactory hairpin structures. A solution of Apt and Apt-C was heated at 90 °C for 10 min and then slowly cooled to 25 °C to form dsDNA (Apt·Apt-C).

### 2.2. Apparatus

**Fig. 1:**
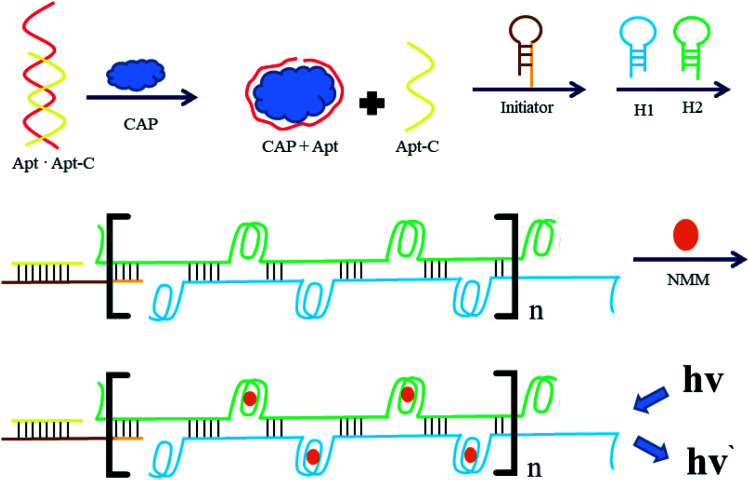
Schematic representation of a label-free fluorescence strategy which was established based on guanine (G)-quadruplex/*N*-methyl mesoporphyrin IX (NMM) complex formation and hybridization chain reaction (HCR) amplification. The “*n*” represents the repeated part of the HCR process.

A pH meter (pHS-3E, Shanghai Lei-ci Instrument Plant, Shanghai, China) was used for pH measurements. Fluorescence measurements were performed at 25 °C using an F-4600 Hitachi fluorescence spectrometer (Tokyo, Japan) equipped with a xenon lamp excitation source. The fluorescence spectra were recorded using a quartz cell with a 1 cm path length, an excitation wavelength at 399 nm, and emission wavelengths of 580–660 nm. The fluorescence intensity at 612 nm was chosen as optimal for evaluating the performance of the proposed assay (Fig. 1S†). The excitation and emission slit widths were both 5 nm.

### 2.3. Fabrication of aptasensor

First, 2.5–200 pg mL^−1^ of CAP were added to a mixture of 1 nmol L^−1^ dsDNA (Apt·Apt-C), 1 nmol L^−1^ initiator DNA, and 4 nmol L^−1^ hairpin DNA probes (H1 and H2), and the solution was kept at 37 °C for 30 min. Subsequently, 1 μmol L^−1^ NMM was added and the mixture was kept in the dark for 10 min. The final mixture was used for fluorescence measurements.

### 2.4. Detection of CAP in real samples

The collection of milk samples was carried out in accordance with the national standards of China (GB4789.18-2010). Then, the proteins in the milk samples were precipitated according to a reported method (Ping *et al.*, 2012). First, 1 mL of spiked milk and 9 mL of PBS were mixed to reduce the matrix effect. Then, the proteins were precipitated by mixing with 1 mL of acetocaustin (15%). Subsequently, the mixture was centrifuged at 5000 rpm for 5 min, and the supernatant was collected and filtered through a 0.22 mm membrane filter to remove residual proteins. The filtrate was transferred to a flask for further analysis and stored in a refrigerator until use.

To evaluate the suitability of the proposed method, the milk samples were spiked with CAP at four different concentrations (25, 50, 100, and 200 ng mL^−1^), and the spiked samples were simultaneously analyzed using the prepared aptasensor and an ELISA kit, which could accurately detect CAP in the concentration range of 25 pg mL^−1^ to 1.5 ng mL^−1^. CAP determination was performed in accordance with the kit's specifications. The CAP determination procedure with the proposed aptasensor was similar to that described in Section 2.3. At each concentration level, three replicates were performed (*N* = 3). The recoveries and relative standard deviations (RSDs) were used to evaluate the accuracy and precision of the method.

## 3. Results and discussion

### 3.1. Principle of proposed aptasensor

The detection principle of the proposed aptasensor is outlined in Fig. 1. In this strategy, based on the base complementary pairing principle, an aptamer for CAP (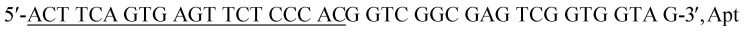) is used as the specific recognition element for the target. In the absence of CAP, the underlined sequence of Apt can partially hybridize with the complementary strand (5′-GT GGG ACA ACT CAC TGA AGT-3′, Apt-C). Apt-C cannot be released from dsDNA (Apt·Apt-C) without the target. However, upon the addition of CAP, owing to the specific binding between Apt and CAP, Apt-C is released from dsDNA, and the amount of released Apt-C depends on the CAP concentration. Then, Apt-C can hybridize with the circular part (underlined sequence) of initiator MB, a hairpin DNA probe (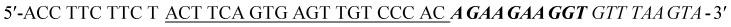), and the hairpin structure (bold sequence) open, releasing a primer sequence (italic sequence) that can trigger HCR between the two complementary hairpin probes (H1 and H2). Once the HCR is triggered, the resulting hybridization cascade forms a nicked double-helix polymer, with stable G-quadruplex DNA structures that can be specifically bound by *N*-methyl mesoporphyrin IX (NMM), and induce an increase in the fluorescence intensity at 612 nm, from this the quantitative determination of CAP was allowed. Conversely, without the target, the hairpin structure of the initiator, H1, and H2 are stable and HCR couldn't trigger, which means there are no signals that could be found.

### 3.2. Feasibility of proposed aptasensor

**Fig. 2:**
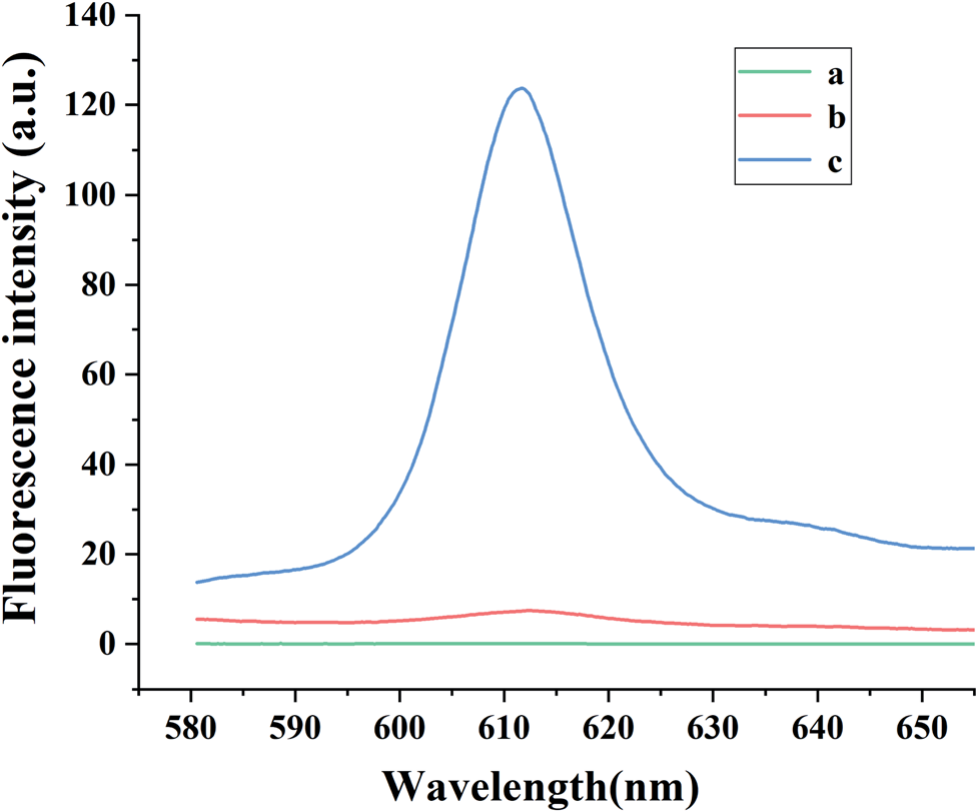
Fluorescence emission spectra of solutions containing various components of the aptasensor: (a) mixture without NMM; (b) mixture without CAP; (c) mixture. Experimental conditions: 200 pg mL^−1^ CAP, 1 nmol L^−1^ Apt·Apt-C, 1 nmol L^−1^ initiator-MB, 4 nmol L^−1^ H1 and H2, and 1 μg mL^−1^ NMM.

To illustrate the viability of this sensing platform, the fluorescence signals were investigated under several conditions. As can be seen in Fig. 2, in the absence of NMM, no emission peak was observed at 612 nm (curve *a*). In the absence of CAP, the HCR cannot be triggered, so G-quadruplexes cannot form and the fluorescence intensity of NMM remains weak (curve *b*). Similar behavior occurred in the absence of Apt·Apt-C, initiator MB or the hairpin probes (H1 and H2). However, with the addition of CAP, the fluorescence intensity at 612 nm increased remarkably (curve *c*), indicating the formation of the HCR product with G-quadruplexes and their complexation with NMM. Furthermore, the systematic quantum yield of G4/NMM was 0.64 (Table S2†).

### 3.3. Optimization of the experimental condition

**Fig. 3:**
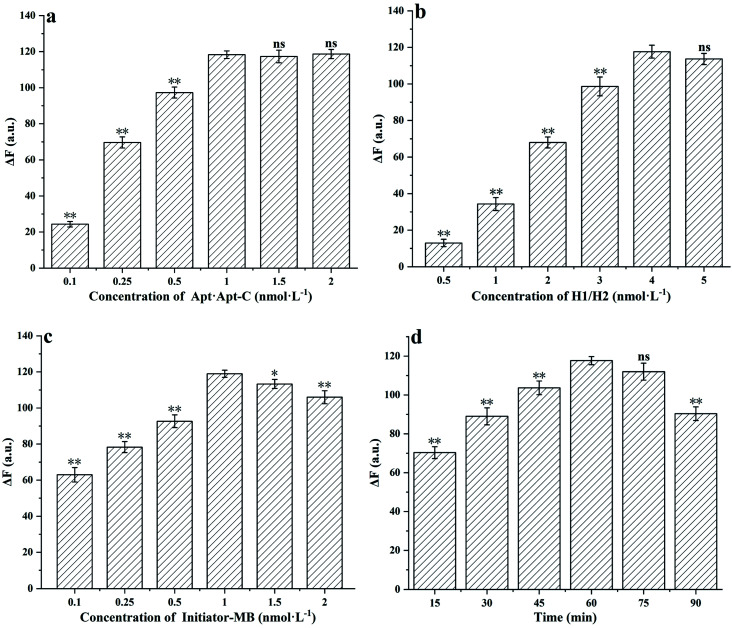
Effects of (a) the concentration of Apt·Apt-C, (b) the concentration of H1/H2, (c) the concentration of initiator MB, (d) the HCR reaction time. The error bars represent the standard deviations of three replicates (*n* = 3, mean ± SD; ns. *p* > 0.05: Compared to the control group, there were no significant differences. *p < 0.05, **p < 0.01: Compared to the control group, there were significant differences. Δ*F* means the changes in the fluorescence intensity between the samples and blank).

To optimize the experimental conditions, the parameters of Apt·Apt-C concentration, H1/H2 concentration, initiator MB concentration and reaction time were tested. And shown as Δ*F*, which means the changes in the fluorescence intensity between the samples and blank. As shown in Fig. 3(a), the Δ*F* increased significantly (*p* < 0.01) with the Apt·Apt-C concentration increasing from 0.1 nmol L^−1^ to 1 nmol L^−1^, the maximum Δ*F* was obtained at the Apt·Apt-C concentration of 1 nmol L^−1^. No significant changes in Δ*F* were observed as the concentration increased from 1 nmol L^−1^ to 2 nmol L^−1^. Hence, 1 nmol L^−1^ was selected as the optimal concentration of Apt·Apt-C for further experiments. The similar situation was also observed in Fig. 3(b), the Δ*F* increased significantly (*p* < 0.01) with the concentration of H1/H2 increasing from 0.5 nmol L^−1^ to 4 nmol L^−1^, and no significant changes in Δ*F* were observed as the concentration was further increased from 4 nmol L^−1^ to 5 nmol L^−1^. Therefore, 4 nmol L^−1^ was selected as the optimal concentration of H1/H2. Fig. 3(c) showed a significant rise of Δ*F* (*p* < 0.01), when the initiator-MB concentration increasing from 0.1 nmol L^−1^ to 1 nmol L^−1^. Increasing initiator-MB concentration beyond 1 nmol L^−1^ resulted in decreased responses of Δ*F*. Therefore, 1 nmol L^−1^ was selected as the optimal initiator-MB concentration for further experiments. In Fig. 3(d), with the HCR reaction time increased to 60 min, the Δ*F* significantly increased and the maximum Δ*F* was observed. And no significant changes in Δ*F*, when the HCR reaction time increased to 75 min. However, the Δ*F* significantly dropped at the 90 min. Consequently, 60 min was selected as the optimal HCR reaction time.

### 3.4. Sensitivity of proposed aptasensor

**Fig. 4:**
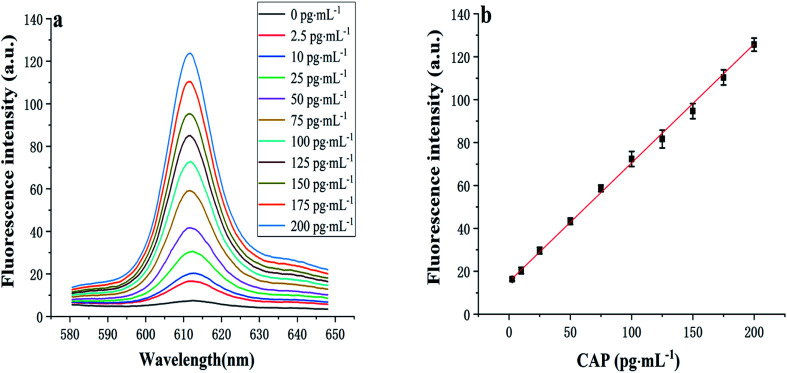
(a) Fluorescence spectra and (b) calibration plot for CAP detection with the developed aptasensor. Experimental conditions: 1 nmol L^−1^ Apt·Apt-C, 1 nmol L^−1^ initiator-MB, 4 nmol L^−1^ H1 and H2, and 1 μg mL^−1^ NMM. Each error bar in (b) represents the standard deviation for three independent experiments.

After determining the optimal conditions (Fig. 2), the sensitivity of the developed aptasensor was investigated. As displayed in Fig. 4, as the CAP concentration increases, the fluorescence intensity at 612 nm gradually increases. The fluorescence intensity was found to be proportional to the CAP concentration in the range of 2.5–200 pg mL^−1^, with a linear regression equation, *I* = 0.55*C* + 15.17 (*R*^2^ = 0.996), where *C* denotes the CAP concentration (pg mL^−1^), *I* represents the fluorescence intensity. The limit of detection (LOD) was determined to be 0.8 pg mL^−1^ (*N* = 3, RSD = 4.7%).

### 3.5. Selectivity of proposed aptasensor

**Fig. 5:**
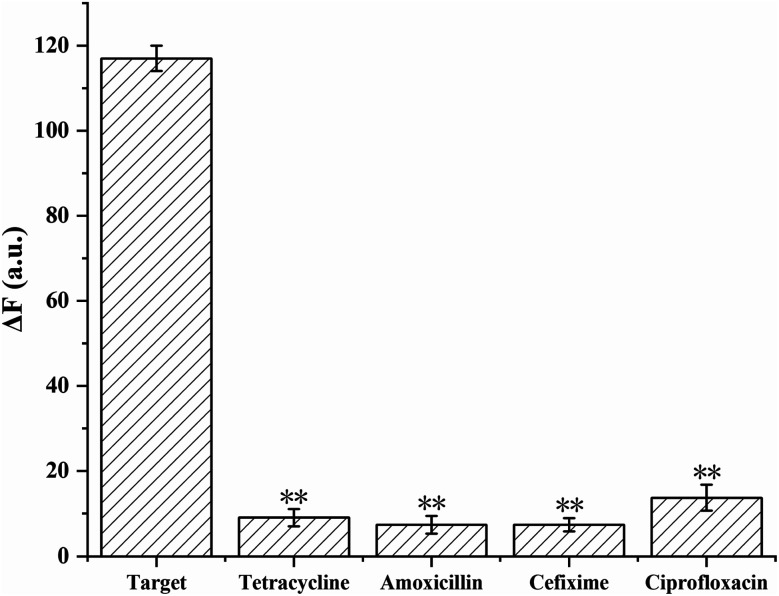
Fluorescence intensity at 612 nm for the detection of CAP and other antibiotics with the developed aptasensor. Experimental conditions: 1 nmol L^−1^ Apt·Apt-C, 1 nmol L^−1^ initiator-MB, 4 nmol L^−1^ H1, and H2, and 1 μg mL^−1^ NMM. The concentration of CAP was 200 pg mL^−1^ CAP, whereas those of the other antibiotics were 2 ng mL^−1^. The error bars denote the standard deviations of three measurements (*n* = 3, mean ± SD, **p < 0.01 *vs.* target group).

To evaluate the selectivity of the proposed method, the aptasensor was separately tested with target and other antibiotics. The target sample was 200 pg mL^−1^ CAP, the other antibiotics samples were 2 ng mL^−1^ ciprofloxacin, 2 ng mL^−1^ tetracycline, 2 ng mL^−1^ cefixime, and 2 ng mL^−1^ amoxicillin. As demonstrated in Fig. 5, compared with other antibiotics, the fluorescence intensity of CAP was significantly higher (*p* < 0.01), which indicated that the proposed aptasensor exhibits high selectivity toward CAP.

### 3.6. Real sample analysis

To verify the feasibility of the aptasensor for directly quantifying CAP in complex biological samples, the aptasensor was applied to the determination of CAP in milk using an ELISA kit as a reference method. As shown in Table 1, the average recoveries with the aptasensor were 99.8–108.3%, and the RSDs were 4.5–5.2%. In contrast, the ELISA kit gave average recoveries and RSDs of 93.0–106.4% and 3.3–8.2%, respectively. These results suggest that the accuracy of the proposed aptasensor is sufficient for the analysis of CAP in real samples and that it possesses good precision. Advantageously, in comparison with the CAP ELISA kits, the developed aptasensor offers increased sensitivity, greater simplicity, and a shorter analysis time.

**Table 1:** Detection of CAP spiked in milk by the proposed method (*n* = 3, mean ± SD)

		The proposed method^a^		The ELISA kit^b^	
Samples	Added (pg mL^−1^)	Recovery (%)	RSD (%)	Recovery (%)	RSD (%)
1	0	Not detected	0	Not detected	0
2	25.0	108.3 ± 5.2	4.8	93.0 ± 7.7	8.2
3	50.0	99.8 ± 4.7	4.7	94.3 ± 6.5	6.9
4	100.0	103.7 ± 4.1	4.5	101 ± 3.4	3.3
5	200.0	100.5 ± 5.3	5.2	106.4 ± 5.7	5.4

a The LOD of the proposed method is 2.5 pg mL^−1^.

b The LOD of the ELISA method is 25 pg mL^−1^.

To clarify the novelty of the aptasensor method developed and validated in this study, its performance parameters were compared with those of other reported methods for CAP determination (Table S3†). Notably, the LOD of the proposed strategy was lower than those of some literature methods. Furthermore, because of the signal amplification provided by the HCR and the specificity between Apt and CAP, the aptasensor method exhibited both high sensitivity and selectivity. Furthermore, because the proposed aptasensor is enzyme-free and homogeneous, it was successfully applied to the analysis of CAP-spiked milk samples. Thus, the proposed sensing platform is expected to be suitable for application in the areas of antibiotic diagnostics and food analysis.

## 4 Conclusion

In this study, a label-free aptasensor for CAP was developed using HCR amplification and G-quadruplex/NMM complexation, and in the real sample-milk detection, it has a good performance with the recoveries of 99.8–108.3% and RSDs of 4.5–5.2%. This method without any label of fluorophores, quenchers, or enzymes, simplifies the detection process and shortens the detection time. In the milk samples detections, the analysis time with the aptasensor was ≤60 min, which is approximately two-fold more rapid than that with ELISA, the conventional method (LOD = 25 pg mL^−1^). Besides, the HCR significantly amplifies the detection signal. After the addition of NMM, the target was transferred to the fluorescence intensity at 612 nm, and a linear detection range of 2.5–200 pg mL^−1^ with a LOD of 0.8 pg mL^−1^ was established, which was allowed to achieve a quantitative evaluation of the CAP and, especially more sensitive than ELISA. Hence, the proposed aptasensor method has the advantages of high sensitivity, good selectivity, and a short detection time, making it favorable potential for antibiotic diagnostics in food, and clinical analysis.

## Abbreviations

CAP: Chloramphenicol
HCR: Hybridization chain reaction
NMM: *N*-Methyl mesoporphyrin IX
ELISA: Enzyme-linked immunosorbent assay
RSD: Relative standard deviation
LOD: Limit of detection

## Conflicts of interest

The authors declare that they have no known competing financial interests or personal relationships that could have appeared to influence the work reported in this paper.

## Supplementary Material

RA-012-D2RA00572G-s001

## References

[cit1] Wang J., Guo J., Zhang J., Zhang W., Zhang Y. (2016). Anal. Methods.

[cit2] Zhang Y., Li H., Xie J., Chen M., Zhang D., Pang P. (2017). J. Electroanal. Chem..

[cit3] Li Y. B., Yuan J. M., Xu Z. X. (2019). J. Anal. Methods Chem..

[cit4] Jiang X., Xu W., Chen X., Liang Y. (2019). Anal. Bioanal. Chem..

[cit5] Wang G., Wang S., Yan C., Bai G., Liu Y. (2018). Colloids Surf., B.

[cit6] Zuo X., Zhang H., Zhu Q., Wang W., Feng J., Chen X. (2016). Biosens. Bioelectron..

[cit7] Choi S., Lee G., Park I. S., Son M., Kim W., Lee H. (2016). Anal. Chem..

[cit8] Zhou W., Ding J., Liu J. (2017). Biosens. Bioelectron..

[cit9] Daşbaşı T., Saçmacı Ş., Ülgen A., Kartal Ş. (2015). J. Ind. Eng. Chem..

[cit10] José A. L., Belén H., Carlos M. (2016). Talanta.

[cit11] Chugaev A. V., Chernyshev I. V. (2012). Geochem. Int..

[cit12] Roman M., Rigo C., Castillo-Michel H., Munivrana I., Vindigni V., Mieti I. (2015). et al.. Anal. Bioanal. Chem..

[cit13] Samira Y., Ali B., Maryam B., Habib N., Dehghan T. M., Mostafa A. (2018). Microchim. Acta.

[cit14] Ebrahimi M., Raoof J. B., Ojani R. (2018). J. Iran. Chem. Soc..

[cit15] Ono A., Torigoe H., Tanaka Y., Okamoto I. (2011). Cheminform.

[cit16] Ono A., Cao S., Togashi H., Tashiro M., Fujimoto T. (2008). Chem. Commun..

[cit17] Liu G., Yuan Y., Wei S., Zhang D. (2014). Electroanalysis.

[cit18] Xi H., Cui M., Li W., Chen Z. (2017). Sens. Actuators, B.

[cit19] Zhang Z., Jing Y. (2014). Sens. Actuators, B.

[cit20] Hou T., Li W., Liu X., Li F. (2015). Anal. Chem..

[cit21] Robert M. D., Niles A. P., Stephen L. M. (2004). Proc. Natl. Acad. Sci. U. S. A..

[cit22] Duan Y., Wang L., Gao Z., Wang H., Zhang H., Li H. (2016). Talanta.

[cit23] Chen J., Zhao Z., Chen Y., Zhang J., Yan L., Zheng X. (2018). et al.. Poult. Sci..

[cit24] Yang L., Tao Y., Yue G., Li R., Qiu B., Guo L. (2016). et al.. Anal. Chem..

[cit25] Teng J., Ye Y., Yao L., Yan C., Cheng K., Xue F. (2017). et al.. Mikrochim. Acta.

[cit26] Wu D., Xu H., Shi H., Li W., Sun M., Wu Z. S. (2016). Anal. Chim. Acta.

[cit27] Zhu D., Zhang L., Ma W., Lu S., Xing X. (2015). Biosens. Bioelectron..

[cit28] Chen Z., Ying L., Chen X., Zhao J., Liu S. (2018). Biosens. Bioelectron..

[cit29] Bao T., Wen M., Wen W., Zhang X., Wang S. (2019). Sens. Actuators, B.

[cit30] Zhang Y., Chen Z., Tao Y., Wang Z., Ren J., Qu X. (2015). Chem. Commun..

[cit31] Lu W., Arumugam S., Senapati D. (2010). ACS Nano.

[cit32] Duan N., Wu S., Chen X., Huang Y., Xia Y., Ma X. (2013). et al.. J. Agric. Food Chem..

[cit33] Wang L., Tan W. (2006). Nano Lett..

[cit34] Ma X., Jiang Y., Jia F., Yu Y., Chen J., Wang Z. (2014). J. Microbiol. Methods.

[cit35] Lian S., Zhang P., Ping G., Hu D., Shi B., Zeng C. (2012). et al.. J. Nanosci. Nanotechnol..

[cit36] Li Y., Liu S., Ling L. (2018). J. Anal. Methods Chem..

[cit37] Li Y., Liu S., Deng Q., Ling L. (2017). J. Med. Virol..

[cit38] Li Y., Ling L. (2015). Microchim. Acta.

[cit39] Li Y., Zhang H., Zhu H., Ling L. (2015). Anal. Methods.

[cit40] Qu L., Dai L. (2005). J. Phys. Chem. B.

[cit41] Zhou Y., Mei L., Su B., Lu Q. (2009). J. Mater. Chem..

[cit42] Lau O. W., Bing S. (2000). Anal. Chim. Acta.

[cit43] Braun G., Lee S. J., Da Nte M., Nguyen T. Q., Reich N. (2007). J. Am. Chem. Soc..

[cit44] Lee J. S., Lytton-Jean A., Hurst S. J., Mirkin C. A. (2007). Nano Lett..

[cit45] Thompson D., Enright A., Faulds K., Smith W., Graham D. (2008). Anal. Chem..

[cit46] Lu S., Hu T., Wang S., Sun J., Yang X. (2016). ACS Appl. Mater. Interfaces.

[cit47] Huo X. L., Zhang N., Xu J. J., Chen H. Y. (2018). Electrochem. Commun..

[cit48] Kong L., Wang D., Chai Y. Q., Yuan Y., Yuan R. (2019). Anal. Chem..

[cit49] Guo X., Liu S., Yang M., Du H., Qu F. (2019). Biosens. Bioelectron..

[cit50] Zhao C., Li W., Ren J., Qu X. (2011). Chem. Commun..

