# Supplementary material for: Label-free fluorescent aptasensor for chloramphenicol based on hybridization chain reaction amplification and G-quadruplex/N-methyl mesoporphyrin IX complexation
Source: RSC Adv. 2022 Jun 22;12(29):18347–53. doi: 10.1039/d2ra00572g (PMC9215126; doi:10.1039/d2ra00572g)
Supplement: RA-012-D2RA00572G-s001 [file RA-012-D2RA00572G-s001.pdf]

Electronic Supplementary Material (ESI) for RSC Advances.
This journal is © The Royal Society of Chemistry 2022

Label-free fluorescence aptasensor for chloramphenicol based on hybridization chain reaction amplification and G-quadruplex/*N*-methyl mesoporphyrin IX complexation

Wentao Zheng [a,#], Yubin Li [b,#], Liting Zhao [b], Ciling Li [b] , Lei Wang [a,*]

[a] Zhanjiang Central Hospital, Guangdong medical university, Zhanjiang, 524045, China.

[b] Faculty of Chemistry & Environmental Science, Guangdong Ocean University, Zhanjiang, 524088, China.

Table S1. Sequences of oligonucleotides

Name	Sequence (5′→3′)
Apt	5′- ACT TCA GTG AGT TGT CCC ACG GTC GGC GAG TCG GTG GTA G - 3′
Apt-C	5′- GT GGG ACA ACT CAC TGA AGT -3′
Initiator-MB	5′- ACC TTC TTC TACT TCA GTG AGT TGT CCC ACA GAA GAA GGT GTT TAA GTA -3′
Hairpin DNA (H1)	5'- AGG GCG GGT GGG TGT TTA AGT TGG AGA ATT GTA CTT AAA CAC CTT CTT CTT GGG T -3′
Hairpin DNA (H2)	5'- TGG GTC AAT TCT CCA ACT TAA ACT AGA AGA AGG TGT TTA AGT TGG GTA GGG CGG G -3'

Spectra properties of samples were detected under the same experimental conditions using an F-4600 Hitachi fluorescence Spectrometer with a xenon lamp. At an excitation of λ = 399nm, the emission peak was recorded at 612 nm both for N-Methyl mesoporphyrin IX(NMM) and NMM/G4 (Figure S1). However, the fluorescence intensity of NMM/G4 was significantly higher than that of NMM, indicating that the NMM/G4 composite greatly enhanced the fluorescence of NMM.

* Corresponding author. E-mail addresses: wangleigdmu@163.com (Lei Wang)

# Wentao Zheng and Yubin Li contributed equally to this work.

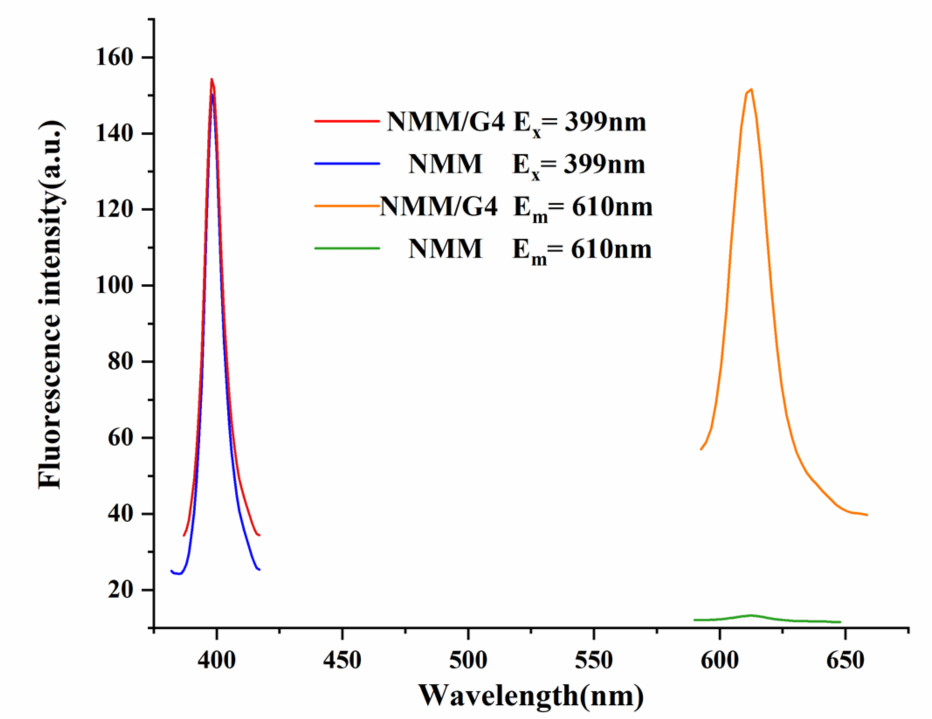

Figure S1. Spectra properties of NMM and NMM/G4

The fluorescence intensity of NMM was enhanced after the complexation reaction with G-quadruplexes. The systematic quantum yield was measured for G4/ NMM, and the quinine sulfate dihydrate which dissolved in 0.1 mol/L H_2SO_4 with a quantum yield of 51% was the reference. The quantum yield (y) is calculated by the following formula.

$$Y = Y_R \cdot \frac{F_s}{F_R} \cdot \frac{A_R}{A_s} \cdot \frac{\eta_s^2}{\eta_R^2}$$

F is the integral area of the fluorescence emission spectrum, A is the absorbance, and η is the refractive index of the solvent (R and S represent the reference quinine sulfate dihydrate solution and the sample respectively)

The systematic quantum yield measurements were shown in table S2.

Table S2. Systematic quantum yield measurements

Test sample		λex/nm	A	F	Y
Reference	quinine sulfate dihydrate	399	0.024	13245	0.51
Sample	G/NMM	399	0.02	15319	0.64

Table S3. Comparison with other methods for CAP detection

Method	Detection limit	Detection range	Ref.
Electrochemiluminescence assay	1.7 $pg\cdot mL^{-1}$	0.01 - 4000 $ng\cdot mL^{-1}$	(Chen et al. 2020)
Structure switching signaling Assay	0.70 ng mL^{-1}	1 - 100 $ng\cdot mL^{-1}$	(Ma et al. 2020)
MOF-based ratiometric fluorescent biosensor	0.08 pg mL^{-1}	0.1pg $\cdot mL^{-1}$ - 10ng$\cdot mL^{-1}$	(Liu et al. 2020)
Electrochemical sensor	0.32 $\mu mol\cdot L^{-1}$	0.03 - 0.5 $mmol\cdot L^{-1}$	(Shad et al. 2019)
Photovoltammetric sensor	0.14 $\mu mol\cdot L^{-1}$	0.5 - 50 $\mu mol\cdot L^{-1}$	(Zhu et al. 2019)
Chemiluminescence resonance energy transfer assay	2.0 $pg\cdot mL^{-1}$	0.01 - 100 $ng\cdot mL^{-1}$	(Jia et al. 2019)
Label free aptasensor based on HCR and G-quadruplex/NMM	0.8 $pg\cdot mL^{-1}$	2.5 - 200 $pg\cdot mL^{-1}$	This work

Chen, J., Zheng, J., Zhao, K., Deng, A., & Li, J. (2020). Electrochemiluminescence resonance energy transfer system between non-toxic SnS2 quantum dots and ultrathin Ag@Au nanosheets for chloramphenicol detection. *Chemical Engineering Journal*, 123670.

Ma, X. Y., Li, H. X., Qiao, S. N., Huang, C. J., Liu, Q. l., & Shen, Xu. (2020). A simple and rapid sensing strategy based on structure-switching signaling aptamers for the sensitive detection of chloramphenicol. *Food Chemistry,* 302, 125359.

Liu, S., Bai, J. L., Huo, Y. P., Ning, B. A., Peng, Y., Li, S., Gao, Z. X. (2020). A zirconium-porphyrin mof-based ratiometric fluorescent biosensor for rapid and ultrasensitive detection of chloramphenicol. *Biosensors and Bioelectronics,* 149, 111801.

Shad, N. A., Bajwa, S. Z., Amin, N., Taj, A., Hameed, S., Khan, Y., Dai, Z., Khan, W.S. (2019). Solution growth of 1D zinc tungstate (ZnWO4) nanowires; design, morphology, and electrochemical sensor fabrication for selective detection of chloramphenicol. *Journal of Hazardous Materials,* 367, 205-214.

Zhu, Y. H., Yan, K., Xu, Z. W., Wu, J. N., & Zhang, J. D. (2019). Cathodic “signal-on” photoelectrochemical aptasensor for chloramphenicol detection using hierarchical porous flower-like Bi-BiOI@C composite. *Biosensors and Bioelectronics,* 131, 79–87.

Jia, B. J., He, X., Cui, P. L., Liu, J. X., & Wang, J. P. (2019). Detection of chloramphenicol in meat with a chemiluminescence resonance energy transfer platform based on molecularly imprinted graphene. *Analytica Chimica Acta ,* 1063, 136-143.
